# Detecting localized damage in cantilevered structures under nonstationary ambient excitations via Gabor spectral mode transmissibility functions

**DOI:** 10.1038/s41598-024-67241-0

**Published:** 2024-07-13

**Authors:** HongJie Zhang, Qigang Sun, DanYu Li, Chen Li, Chunhui He, Gang Liu

**Affiliations:** 1 Power Transmission and Transformation Engineering Department, China Electric Power Research Institute, Beijing, 100192 China; 2 Economic and Technology Research Institute, State Grid Shandong Electric Power Company, Jinan, 250021 China; 3 School of Civil Engineering, Chongqing University, Chongqing, 400045 China

**Keywords:** Damage detection, Nonstationary excitations, Mode transmissibility function, Gabor transform, Singular value decomposition, Civil engineering, Computational science

## Abstract

A method based on Gabor spectral mode transmissibility functions (GSMTFs) is proposed to detect local damage in a cantilevered structure under nonstationary ambient excitations. Gabor transformation and singular value decomposition are used to reduce the influences of other vibration modes on Gabor spectral mode transmissibility functions and process nonstationary structural responses, respectively. A new state characteristic based on the fundamental structure frequency is formulated on the basis of the GSMTFs, eventually leading to the development of a new damage indicator. The probability density functions of the damage indicator for healthy and damaged states can be estimated from the measured data, and the receiver operating characteristic (ROC) curve derived from these probability distributions and the corresponding area under the ROC curve (AUC) are used to determine the damage location. A six-degree-of-freedom system and a typical transmission tower are numerically studied, and the results show that the proposed method can estimate the structural damage location under nonstationary random loads. The proposed method is further validated with a planar frame in the laboratory, which exhibits multiple damage elements via random force hammer excitations. The results show that the AUC values computed for certain parts of the structure containing the damaged elements are greater than those for other parts of the structure, indicating the effectiveness of the proposed method. Moreover, the proposed method is compared with the dot product difference (DPD) index, and the results from the laboratory planar frame demonstrate that the proposed method can better identify damage.

## Introduction

Owing to material degradation, recurring seismic excitations, and wind loads, civil engineering structures inevitably suffer different types of damage, resulting in a gradual degeneration of structural integrity, as indicated by the one-third of bridges in the United States that need to be repaired^1^. Therefore, the early detection of structural damage is essential for ensuring the safe operation of structures. The characteristics of structural responses inevitably change after damage occurs. Many damage detection methods based on measured structural responses have been developed in the last three decades^2–6^, and they can be categorized into three groups: frequency, time, and time‒frequency domain methods.

The frequency domain methods use the measured dynamic features of the structure for damage diagnosis, such as the natural frequencies, mode shapes and transmissibility functions (TFs)^7–9^. These methods have a clear physical meaning and have been shown to be successful for systems under ambient excitations^10–13^. The natural frequencies can be used to establish the natural frequency vector assurance criterion (NFVAC), which can be used to update the finite element model (FEM) and identify damage^14^. Ghannadi P. and Kourehli S.S. proposed structural damage detection methods based on expanded mode shapes and artificial neural networks, which have been successfully applied to truss structures and cantilevered beams^15,16^. By combining natural frequencies and mode shapes, several new objective functions have been proposed^17^, and optimization algorithms have been used to identify structural damage^18^. Worden et al.^19^ proposed TFs as the damage metric from measured white noise excitations, and the saw-cut damage in a stringer was successfully identified. Simon Chesné et al.^20^ employed TFs to detect and localize element stiffness loss in a four-degree-of-freedom (DoF) spring‒mass system and a simulated cantilevered beam. TFs, among all the dynamic features reported, have shown promising sensitivity to structural damage. However, TF algorithms are subject to the effects of the excitation location, the selection of the analysis frequency bandwidth and the environmental noise effect.

Stationary white ambient excitation was assumed in the above studies. Wang et al.^21^ adopted power spectral density functions to evaluate TFs, and their results revealed that the identification error of the lower mode order is small under white noise excitations. Iván Gómez Araújo et al.^22^ proposed an operational modal analysis method based on power spectral density transmissibility matrices. It was demonstrated that the TFs at the natural frequencies converge to a ratio of modal vibration amplitudes. Mao^23^ adopted TFs at the fundamental frequency of a 3-DoF spring–mass system to identify structural damage under stationary excitations. TFs at the fundamental frequency of a structure have been shown to be sensitive to local damage.

Ambient excitations from vehicles and wind loads are nonstationary^24^. Jun Luo et al.^25^ proposed the use of wavelet spectral transmissibility functions to address the damage detection problem of structures under these excitations with a damage indicator. Nevertheless, the study was confined to a shear structure, and the effect of measurement noise was not considered. Furthermore, the selection of wavelet basis functions for the wavelet transform is subjective. The Gabor transform has a high time‒frequency resolution, with the product of the temporal resolution and frequency resolution equal to 0.5. It is a popular time‒frequency analysis method, and selection criteria for shape parameters in the Gabor transform have been proposed^26^. Recently, the Gabor transform and corresponding Gabor spectral mode transmissibility functions (GSMTFs) have been successfully applied to mode shape identification under stationary white noise excitation, with singular value decomposition (SVD) used to reduce the influence of other vibration modes on the Gabor coefficient of modal responses at natural frequencies^26^. The error of identification for the lower mode order is smaller, and the GSMTFs at the natural frequencies converge to a ratio of amplitudes of vibration modes.

The Gabor transform and the GSMTFs at the fundamental frequency of the structure are introduced briefly for handling the nonstationary responses used to construct a new damage indicator for detecting damage in cantilever structures. The Gabor transformation and SVD are used to process the nonstationary dynamic responses and reduce the influence of other vibration modes on the Gabor coefficient of responses at natural frequencies^26^. The existing TF calculation methods are mostly based on power spectrum estimation, which cannot be used for nonstationary responses in theory. As a commonly used nonstationary signal processing method, the Gabor transform can avoid these disadvantages. The damage indicator is derived on the basis of the GSMTFs at the fundamental frequency. The ROC curve and the area under the ROC curve (AUC) are used to localize the damage. Two numerical examples and a laboratory structure are assessed to demonstrate the feasibility of the proposed algorithm.

The rest of this paper is organized as follows. Section “Basic theory of the damage detection method based on GSMTFs” presents the basic theory of the damage detection method, which is based on GSMTFs, followed by the derivation of the new damage indicator. Section “Numerical validation” validates the proposed approach with two numerical examples. Section “Laboratory verification” presents the experimental validation of the proposed approach with a laboratory frame structure. The results are compared with those obtained from the dot product difference (DPD) index. Conclusions are presented in Section “Conclusions”.

## Basic theory of the damage detection method based on GSMTFs

### Purification of GSMTFs via the Gabor transform and SVD

For a given signal *y*(*t*), its Gabor coefficients can be expressed as^27^

1 $$ G_{y} \left( {b,a} \right) = \sum\limits_{{k_{1} = 0}}^{M - 1} {y\left( {k_{1} } \right)g^{*} \left( {k_{1} - bN} \right)e^{{ - i\frac{2\pi }{M}k_{1} a}} } , $$

where * denotes the complex conjugate, *g* is the basis function, *b* is the time shift, *a* is the frequency shift, *N* is the length of the window function and *M* is the number of discrete frequency points. A Gaussian window function is usually used as the basis function. The time-domain and frequency-domain expressions of the Gaussian window function can be expressed as

2 $$ g\left( \tau \right) = \frac{1}{{\sqrt {2\pi } \sigma }}e^{{{{ - \tau^{2} } \mathord{\left/ {\vphantom {{ - \tau^{2} } {2\sigma^{2} }}} \right. \kern-0pt} {2\sigma^{2} }}}} \begin{array}{*{20}c} {} & , \\ \end{array} \quad g\left( \omega \right) = e^{{{{ - \sigma^{2} \omega^{2} } \mathord{\left/ {\vphantom {{ - \sigma^{2} \omega^{2} } 2}} \right. \kern-0pt} 2}}} , $$

where σ is the shape parameter of the Gaussian window function, which affects the frequency resolution and temporal resolution of the basis function and can be determined from the literature^26^.

For an *n*-DoF structure, the Gabor coefficients of the acceleration response at the *i-th* DOF, i.e., signal *ÿ*_*i*_, can be expressed as

3 $$ \begin{gathered} G_{{\ddot{y}_{i} }} \left( {b,a} \right) = \sum\limits_{{k_{1} = 0}}^{M - 1} {\ddot{y}_{i} \left( {k_{1} } \right)g^{*} \left( {k_{1} - bN} \right)e^{{ - i\frac{2\pi }{M}k_{1} a}} } = \sum\limits_{r = 1}^{n} {\frac{{\omega_{r} \phi_{ir} }}{{m_{r} \sqrt {1 - \xi_{r}^{2} } }}D_{r} \left( {b,a_{r} } \right)} \hfill \\ D_{r} \left( {b,a_{r} } \right) = \sum\limits_{{k_{2} { = }0}}^{ + \infty } {\left\{ {e^{{ - \xi_{r} \omega_{r} k_{2} \Delta t}} \cos (\omega_{dr} k_{2} \Delta t + \theta_{r} )G\left( {u,b,a_{r} } \right)} \right\}} \hfill \\ G\left( {u,b,a_{r} } \right) = \sum\limits_{g = 1}^{n} {\phi_{gr} \left( {\sum\limits_{{k_{1} = 0}}^{M - 1} {u_{g} \left( {k_{1} - k_{2} } \right)g^{*} \left( {k_{1} - bN} \right)e^{{ - i\frac{2\pi }{M}k_{1} a_{r} }} } } \right)} \hfill \\ \end{gathered} $$

where *ω*_*r*_ is the *r-th* modal frequency, *ϕ*_*ir*_ is the modal amplitude of the *r*-*th* mode, *m*_*r*_ and *ξ*_*r*_ are the modal mass and modal damping ratio of the *r-th* mode, *a*_*r*_ is the frequency shift corresponding to the *r-th* modal frequency, and *ω*_*dr*_ is the *r-th* damped modal frequency.

The *r-th* modal response is usually assumed to be dominated by that from the corresponding vibration mode with the approaches in Eq. (3). Therefore, the Gabor coefficients of signal *ÿ*_*i*_ at the *r-th* modal frequency can be expressed as

4 $$ \begin{gathered} G_{{\ddot{y}_{i} }} \left( {b,a \to a_{r} } \right) = G_{{\ddot{y}_{i,r} }} \left( {b,a_{r} } \right) + \Delta G_{{\ddot{y}_{i,r} }} \left( {b,a_{r} } \right) \hfill \\ G_{{\ddot{y}_{i,r} }} \left( {b,a_{r} } \right) = \frac{{\phi_{ir} \omega_{r} }}{{m_{r} \sqrt {1 - \xi_{r}^{2} } }}D_{r} \left( {b,a_{r} } \right) \hfill \\ \Delta G_{{\ddot{y}_{i,r} }} \left( {b,a_{r} } \right) = \sum\limits_{\begin{subarray}{l} k = 1 \\ k \ne r \end{subarray} }^{n} {\frac{{\omega_{k} \phi_{ik} }}{{m_{k} \sqrt {1 - \xi_{k}^{2} } }}D_{k} \left( {b,a_{k} } \right)} \hfill \\ \end{gathered} $$

where $G_{{\ddot{y}_{i,r} }} \left( {b,a_{r} } \right)$ and $\Delta G_{{\ddot{y}_{i,r} }} \left( {b,a_{r} } \right)$ are the Gabor coefficients of the *r-th* vibration mode and other vibration modes, respectively.

Furthermore, to reduce the influence of other vibration modes in the *r-th* modal response, a Gabor coefficient matrix **H**(*a*_*r*_) is constructed, and SVD^28,29^ is used to extract the purified matrix ***Ĥ***(*a*_*r*_)^26^, which is related only to the *r-th* modal response using the first left and right singular vectors, as shown below.

5 $$ \begin{gathered} {\mathbf{H}}\left( {a_{r} } \right)\, = \left[ {\begin{array}{*{20}c} {G_{{\ddot{y}_{1} }} \left( {b_{1} ,a_{r} } \right)} & {G_{{\ddot{y}_{1} }} \left( {b_{2} ,a_{r} } \right)} & \cdots & {G_{{\ddot{y}_{1} }} \left( {b_{L} ,a_{r} } \right)} \\ {G_{{\ddot{y}_{2} }} \left( {b_{1} ,a_{r} } \right)} & {G_{{\ddot{y}_{2} }} \left( {b_{2} ,a_{r} } \right)} & \cdots & {G_{{\ddot{y}_{2} }} \left( {b_{L} ,a_{r} } \right)} \\ \vdots & \vdots & \ddots & \vdots \\ {G_{{\ddot{y}_{n} }} \left( {b_{1} ,a_{r} } \right)} & {G_{{\ddot{y}_{n} }} \left( {b_{2} ,a_{r} } \right)} & \cdots & {G_{{\ddot{y}_{n} }} \left( {b_{L} ,a_{r} } \right)} \\ \end{array} } \right] \hfill \\ \quad \quad \;\; = \underbrace {{\left[ {\begin{array}{*{20}c} {\hat{G}_{{\ddot{y}_{1,r} }} \left( {b_{1} ,a_{r} } \right)} & {\hat{G}_{{\ddot{y}_{1,r} }} \left( {b_{2} ,a_{r} } \right)} & \cdots & {\hat{G}_{{\ddot{y}_{1,r} }} \left( {b_{L} ,a_{r} } \right)} \\ {\hat{G}_{{\ddot{y}_{2,r} }} \left( {b_{1} ,a_{r} } \right)} & {\hat{G}_{{\ddot{y}_{2,r} }} \left( {b_{2} ,a_{r} } \right)} & \cdots & {\hat{G}_{{\ddot{y}_{2,r} }} \left( {b_{L} ,a_{r} } \right)} \\ \vdots & \vdots & \ddots & \vdots \\ {\hat{G}_{{\ddot{y}_{n,r} }} \left( {b_{1} ,a_{r} } \right)} & {\hat{G}_{{\ddot{y}_{n,r} }} \left( {b_{2} ,a_{r} } \right)} & \cdots & {\hat{G}_{{\ddot{y}_{n,r} }} \left( {b_{L} ,a_{r} } \right)} \\ \end{array} } \right]}}_{{{\hat{\mathbf{H}}}{ = }s_{1} u_{1} v_{1}^{T} }} + \underbrace {{\,\left[ {\begin{array}{*{20}c} {\Delta \hat{G}_{{\ddot{y}_{1,r} }} \left( {b_{1} ,a_{r} } \right)} & {\Delta \hat{G}_{{\ddot{y}_{1,r} }} \left( {b_{2} ,a_{r} } \right)} & \cdots & {\Delta \hat{G}_{{\ddot{y}_{1,r} }} \left( {b_{L} ,a_{r} } \right)} \\ {\Delta \hat{G}_{{\ddot{y}_{2,r} }} \left( {b_{1} ,a_{r} } \right)} & {\Delta \hat{G}_{{\ddot{y}_{2,r} }} \left( {b_{2} ,a_{r} } \right)} & \cdots & {\Delta \hat{G}_{{\ddot{y}_{2,r} }} \left( {b_{L} ,a_{r} } \right)} \\ \vdots & \vdots & \ddots & \vdots \\ {\Delta \hat{G}_{{\ddot{y}_{n,r} }} \left( {b_{1} ,a_{r} } \right)} & {\Delta \hat{G}_{{\ddot{y}_{n,r} }} \left( {b_{2} ,a_{r} } \right)} & \cdots & {\Delta \hat{G}_{{\ddot{y}_{n,r} }} \left( {b_{L} ,a_{r} } \right)} \\ \end{array} } \right]}}_{{\Delta {\hat{\mathbf{G}}}{ = }s_{2} u_{2} v_{2}^{T} + \cdots + s_{n} u_{n} v_{n}^{T} }} \hfill \\ \end{gathered} $$

where ***Ĥ*** and Δ***Ĝ*** denote the singular value decomposed Gabor coefficient matrices of the *r-th* vibration mode and other vibration modes of signal *ÿ*_*i*_, respectively. ***s***_***i***_, ***u***_***i***_ and ***v***_***i***_ are the *i-th* singular values, *i-th* left singular column vectors, and *i-th* right singular column vectors of matrix **H**(*a*_*r*_), respectively. *L* is the length of signal *ÿ*_*i*_. *b*_*e*_ (*e* = 1,2,…,*L*) denotes the *e*-*th* time shift. Hence, the GSMTFs between the *i-th* and *j-th* DoFs with a *k-th* reference DoF can be defined via the purified Gabor coefficients of the modal responses in matrix ***Ĥ***(*a*_*r*_) as^26^

6 $$ GSMTF_{i,j}^{k} \left( {a_{r} } \right) = \frac{{\sum\limits_{e = 1}^{L} {\hat{G}_{{\ddot{y}_{i,r} }} \left( {b_{e} ,a_{r} } \right)\hat{G}^{ * }_{{\ddot{y}_{k,r} }} \left( {b_{e} ,a_{r} } \right)} }}{{\sum\limits_{e = 1}^{L} {\hat{G}_{{\ddot{y}_{j,r} }} \left( {b_{e} ,a_{r} } \right)\hat{G}^{ * }_{{\ddot{y}_{k,r} }} \left( {b_{e} ,a_{r} } \right)} }} = \frac{{\phi_{ir} }}{{\phi_{jr} }}, $$

where * denotes the conjugate. GSMTF^*k*^_*i*,*i*-1_ can be calculated via the purified Gabor coefficients of the modal responses *ÿ*_*i*,*r*_, *ÿ*_*j*,*r*_ and *ÿ*_*k*,*r*_ extracted from the purified Gabor coefficient matrices in Eq. (5). The purified Gabor coefficient matrices are obtained from all measured responses of the whole structure. As shown in Eq. (6), the GSMTF^*k*^_*i*,*i*-1_(*a*_*r*_) is equal to mode shape coefficient ratios *ϕ*_*ir*_/*ϕ*_*jr*_ in theory. However, if the lower and higher mode contributions are considered, because of the influence of $\Delta G_{{\ddot{y}_{i,r} }} \left( {b,a_{r} } \right)$, the GSMTF^*k*^_*i*,*i*-1_(*a*_*r*_) cannot be derived into the ratios *ϕ*_*ir*_/*ϕ*_*jr*_ in theory, and the established damage indicator does not have a clear physical meaning.

### The proposed damage indicator

After the structural natural frequencies are identified, the purified GSMTFs at the fundamental frequency are selected to form the damage indicator because they can be identified more accurately under ambient excitations. The reason for using the GSMTFs at the fundamental frequency is that there is a corresponding relationship between the proposed *D*_*i*,*i*+1_ (*i* = 1,2,…,*n*-1) and the curvature value of the mode shape, and the calculation of curvature values is prone to significant errors at the location where the mode coefficient symbol changes, which is more likely to appear in the second and upper modal orders.

**Figure 1:**
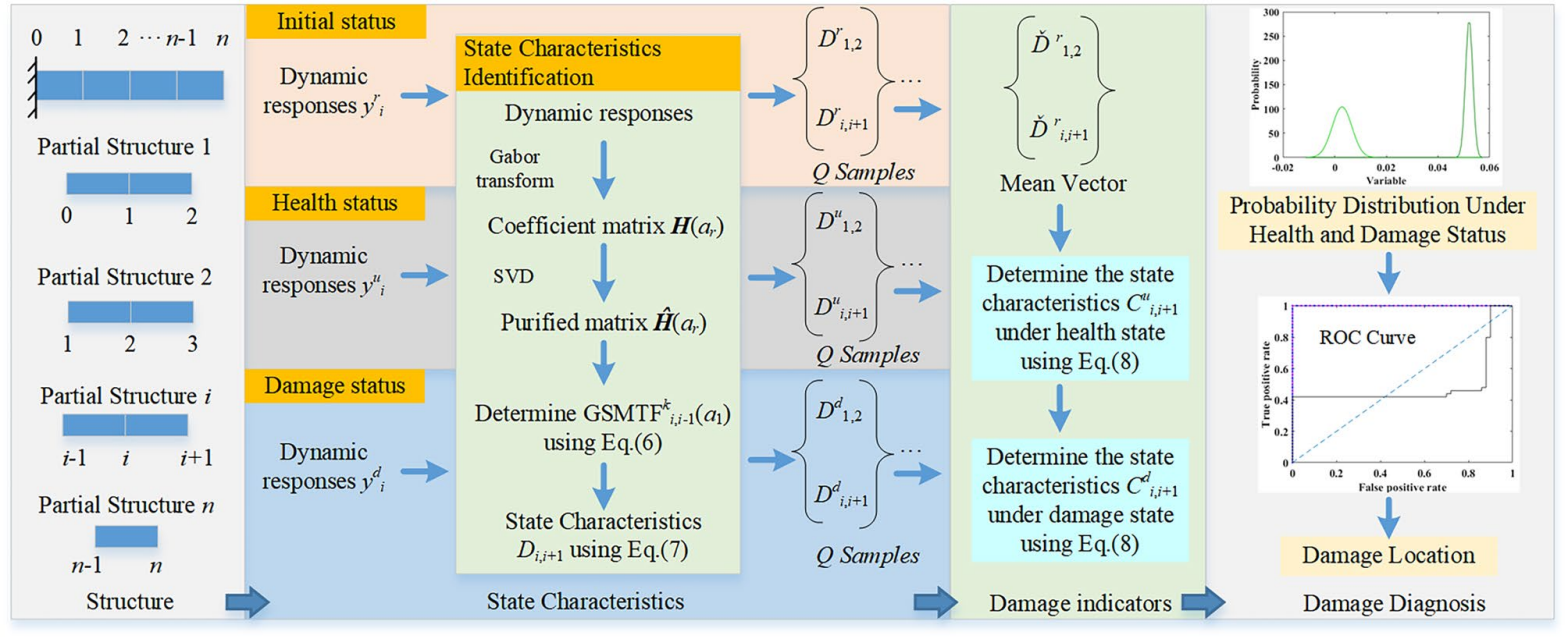
Flowchart of damage diagnosis using purified GSMTFs of the fundamental frequency.

Taking a structure with *n* elements, as shown in Fig. 1, the state characteristics associated with the modal curvature of two adjacent connected elements can be defined as

7 $$ \left\{ \begin{gathered} D_{1,2} = - 2 + \frac{1}{n}\sum\limits_{k = 1}^{n} {GSMTF_{2,1}^{k} \left( {a_{1} } \right)} = - 2 + \frac{{\phi_{2,1} }}{{\phi_{1,1} }} \hfill \\ D_{i,i + 1} = 1 - 2\frac{1}{n}\sum\limits_{k = 1}^{n} {GSMTF_{i,i - 1}^{k} \left( {a_{1} } \right)} + \frac{1}{n}\sum\limits_{k = 1}^{n} {GSMTF_{i + 1,i - 1}^{k} \left( {a_{1} } \right)} = 1 - 2\frac{{\phi_{i,1} }}{{\phi_{i - 1,1} }} + \frac{{\phi_{i + 1,1} }}{{\phi_{i - 1,1} }}\quad \left( {i = 2,3, \cdots ,n - 1} \right) \hfill \\ \end{gathered} \right., $$

where *k* is the reference DoF. Any measured DOF in a structure can be used as the reference DoF, and the mean of GSMTF^*k*^_*i*+1,*i*-1_ of the Gabor spectral mode transmissibility functions between two DoFs can be calculated; subsequently, the state characteristics are *D*_*i*,*i*+1_. As shown in Eq. (7), there is a corresponding relationship between *D*_*i*,*i*+1_ (*i* = 1,2,…,*n-*1) and the first-order mode shape at nodes in elements *i* and *i* + 1, as shown below. *D*_*i*,*i*+1_ represents the curvature value of the first-order mode shape at nodes in elements *i* and *i* + 1. Therefore, *D*_*i*,*i*+1_ can be used to characterize the healthy state of elements *i* and *i* + 1, and the changes in the state characteristics *D*_*i*,*i*+1_ under the initial state and damage state can be used to identify damage.

Since there are errors from measurement and computation, the calculated state characteristics *D*_*i*,*i*+1_ vary, even if the structure is not damaged. To reduce the influence of the above errors on damage identification, three structural states are defined, i.e., the initial state, healthy state and damaged state. The corresponding state characteristics can be expressed as *D*^*r*^_*i*,*i*+1_, *D*^*u*^_*i*,*i*+1_ and *D*^*d*^_*i*,*i*+1_. *D*^*r*^_*i*,*i*+1_ and *D*^*u*^_*i*,*i*+1_ are both calculated from the responses under undamaged structures. If *Q* samples of responses of the structure under initial, healthy and damaged states are collected, *Q* samples of indices *D*^*r*^_*i*,*i*+1_, *D*^*u*^_*i*,*i*+1_ and *D*^*d*^_*i*,*i*+1_ will be obtained. Then, the mean value of *D*^*r*^_*i*,*i*+1_ can be calculated and used as the final identified index under the initial state, which can be expressed as *Ď*^*r*^_*i*,*i*+1._ Finally, the damage indicators *C*^*u*^_*i*,*i*+1_ and *C*^*d*^_*i*,*i*+1_ of the Q samples can be calculated as follows:

8 $$ \begin{gathered} C_{i,i + 1}^{u} = \left| {D_{i,i + 1}^{u} - \check{D}_{i,i + 1}^{r} } \right|\begin{array}{*{20}c} {} & {\left( {i = 1,2, \cdots ,n - 1} \right)} \\ \end{array} \hfill \\ C_{i,i + 1}^{d} = \left| {D_{i,i + 1}^{d} - \check{D}_{i,i + 1}^{r} } \right|\begin{array}{*{20}c} {} & {\left( {i = 1,2, \cdots ,n - 1} \right)} \\ \end{array} \hfill \\ \end{gathered} $$

where *C*^*u*^_*i*,*i*+1_ and *C*^*d*^_*i*,*i*+1_ are the damage indicators for the pair of elements *i* and *i* + 1 under healthy and damaged states, respectively. GSMTF, D and C are matrices or vectors, and GSMTF^*k*^_*i*,*i*-1_, D_*i*,*i*+1_ and C_*i*,*i*+1_ are scalers.

Furthermore, the probability density functions (PDFs) of the damage indicators *C*^*u*^_*i*,*i*+1_ and *C*^*d*^_*i*,*i*+1_ are assumed to be normally distributed as

9 $$ \begin{gathered} y_{u} = f\left( {C_{i,i + 1}^{u} |\mu_{u} ,p_{u} } \right) = \frac{1}{{p_{u} \sqrt {2\pi } }}e^{{\frac{{ - \left( {C_{i,i + 1}^{u} - \mu_{u} } \right)^{2} }}{{2p_{u}^{2} }}}} \hfill \\ y_{d} = f\left( {C_{i,i + 1}^{d} |\mu_{d} ,p_{d} } \right) = \frac{1}{{p_{d} \sqrt {2\pi } }}e^{{\frac{{ - \left( {C_{i,i + 1}^{d} - \mu_{d} } \right)^{2} }}{{2p_{d}^{2} }}}} \hfill \\ \end{gathered} $$

where subscripts *u* and* d* denote the healthy state and damaged state, respectively. *μ*_*u*_ and *p*_*u*_ are the mean and standard deviation, respectively, of *C*^*u*^_*i*,*i*+1_, where *μ*_*d*_ and *p*_*d*_ are the mean and standard deviation of *C*^*d*^_*i*,*i*+1_, respectively.

Therefore, the proposed damage detection method is feasible because it checks whether the PDFs of *C*^*u*^_*i*,*i*+1_ and *C*^*d*^_*i*,*i*+1_ have been changed, as noted from the ROC curve and AUC values. The ROC curve^30^ can then be drawn from the PDFs for the healthy and damaged states of the structure, and the AUC is used to determine the damage location. The AUC has a maximum value of unity, which corresponds to a complete damage state, and a value of 0.5 corresponds to an undamaged state of the pair of elements considered. More detailed discussions on the theory of AUCs and ROCs can be found in^30^.

The flowchart of damage diagnosis of the proposed algorithm is shown in Fig. 1 and described below.

1. The index *D*^*r*^_*i*,*i*+1_ for the intact structure is calculated from the purified GSMTFs. Q samples of the responses of the structure under nonstationary excitations are collected, and the recommended value of Q should be greater than 33 for good curve fitting^31^ of the damage indicator PDFs in Eq. (9). The value of Q is determined on the basis of the total length of the data and should be as large as possible. For each sample of responses, the Gabor coefficients of the signals are calculated with the matrix **H**(*a*_*r*_) obtained from Eq. (5). The purified GSMTFs and the state characteristics *D*^*r*^_*i*,*i*+1_ are obtained from Eqs. (6) and (7), respectively. One state characteristic *D*^*r*^_*i*,*i*+1_ is obtained from one sample of response data. Therefore, Q samples of state characteristics *D*^*r*^_*i*,*i*+1_ can be obtained. Similarly, Q samples of indices *D*^*u*^_*i*,*i*+1_ and *D*^*d*^_*i*,*i*+1_ under healthy and damage states are similarly calculated.
2. The mean value of *D*^*r*^_*i*,*i*+1_ is then calculated, and the damage indicators *C*^*u*^_*i*,*i*+1_ and C^*d*^_*i*,*i*+1_ can be obtained from Eq. (8).
3. The PDFs of damage indicators *C*^*u*^_*i*,*i*+1_ and C^*d*^_*i*,*i*+1_ are obtained from the estimated AUC to localize the damage.

## Numerical validation

### A 6-DoF spring‒mass system

#### Damage scenarios studied and nonstationary excitations

**Figure 2:**
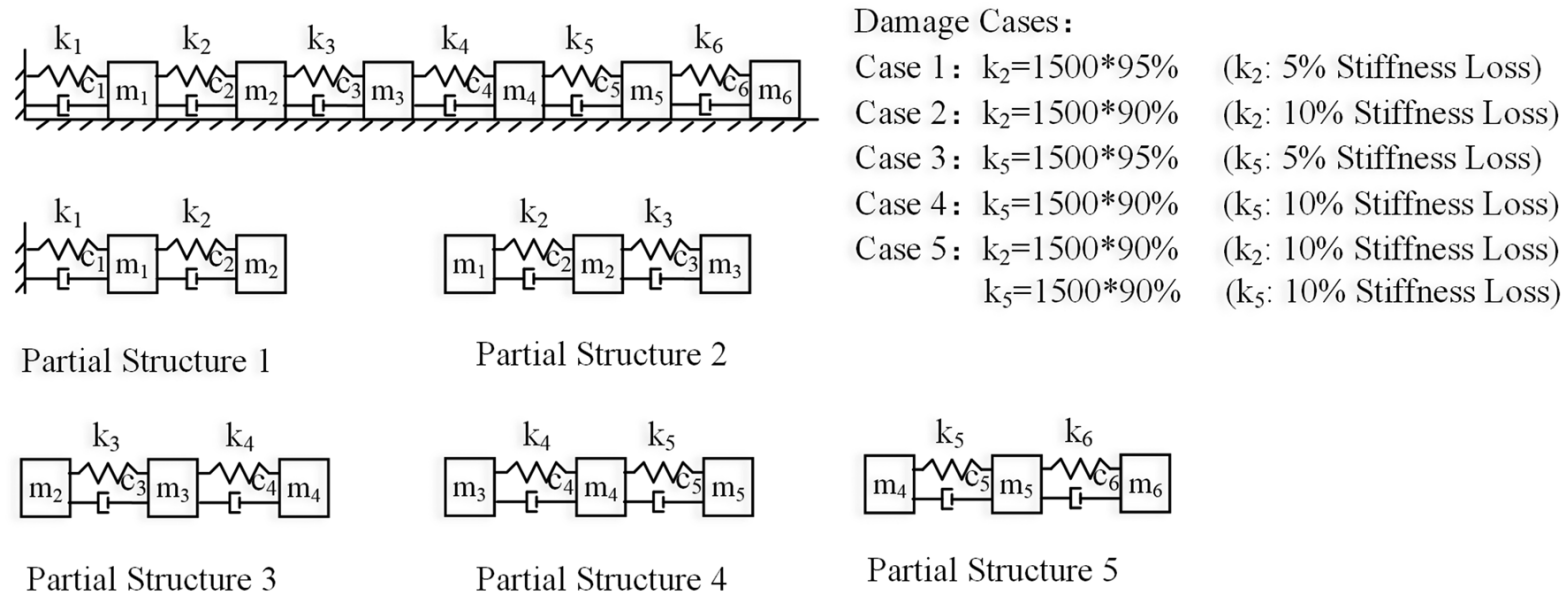
The 6-DoF system and damage scenarios.

A 6-DoF spring‒mass system, as shown in Fig. 2, is studied. The weights of all masses are equal to 1.0 kg, and the spring stiffnesses are all equal to 1500 N/m. Proportional damping is assumed with **[*C*]** = *α***[*M*]** + *β***[*K*]**, *α* = 0.3081 and *β* = 7.5 × 10^–4^. Local damage to the structure is simulated by reducing the spring stiffness values, and the five damage scenarios studied are shown in Fig. 2.

A horizontal nonstationary force is applied to mass 6 without loss of generality. It is generated from the filtering of white noise via an amplitude modulation function as follows:

10 $$ A\left( t \right) = e^{ - 0.001t} - e^{ - 0.002t} $$

The horizontal acceleration responses at all the DoFs under the applied force are calculated via the Newmark method with a sampling frequency of 50 Hz for a period of 1000 s. Fifty sets of structural responses for the intact and healthy states of the structure and when the structure has one of the damage scenarios shown in Fig. 2 are similarly obtained.

**Figure 3:**
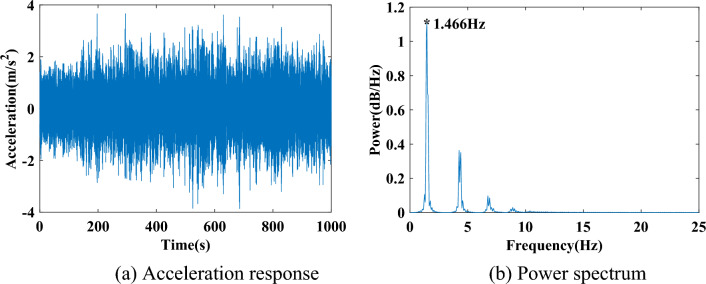
Acceleration response and power spectrum of mass 6 without noise.

The response of mass 6 without noise is shown in Fig. 3 together with its power spectral diagram for illustration. The fundamental frequency of the system is noted at 1.466 Hz. The power spectrum is estimated via the modified periodogram method. The number of discrete Fourier transform points is 1024, and the Hanning window is used. This signal processing technique is adopted in all the studies in this paper.

#### Damage detection without noise

**Figure 4:**
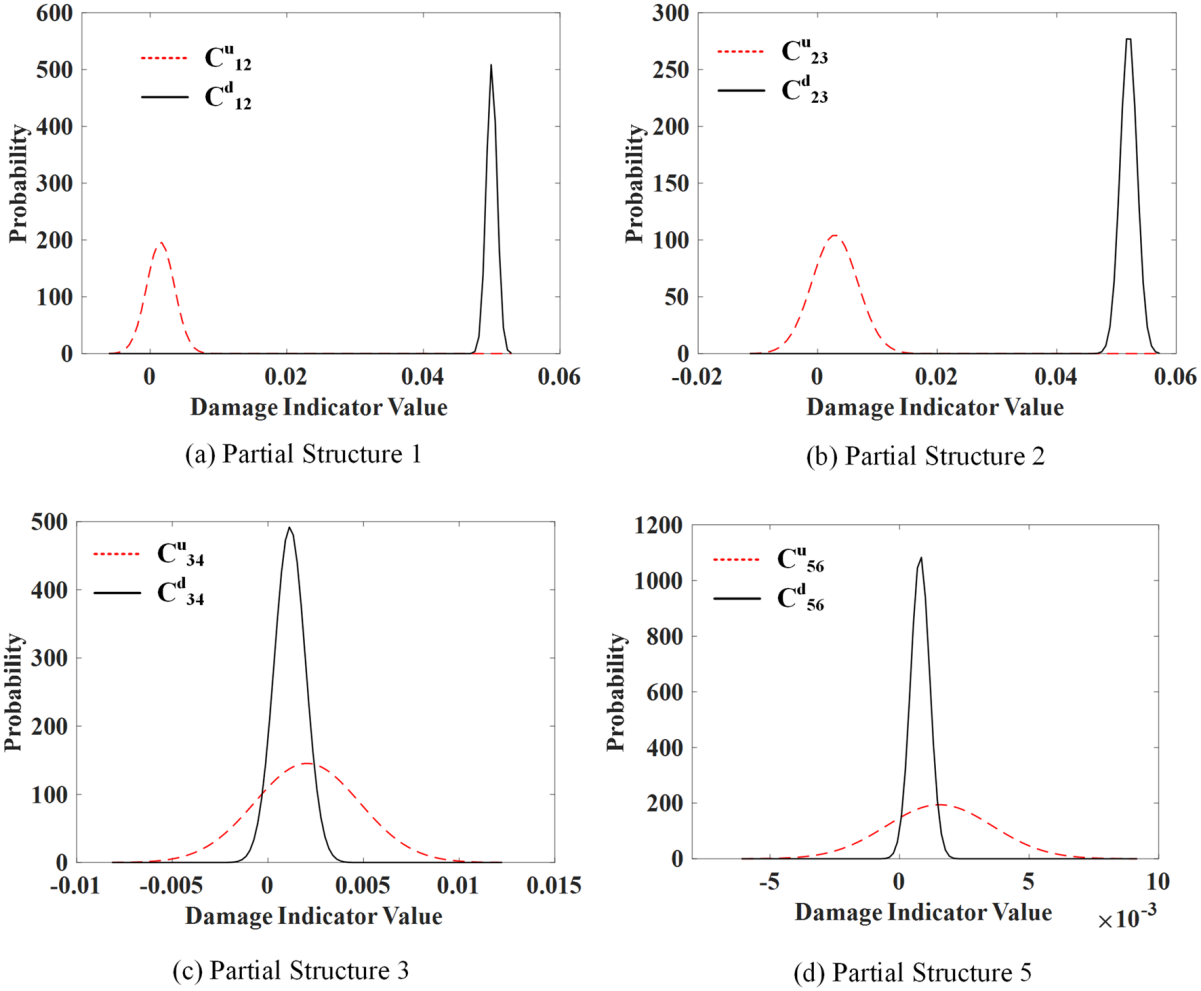
PDFs of the damage indicators under damage case 1.

The number of discrete points in the Gabor transform is 1024, with a shape parameter of 2.306. The purified GSMTFs at the fundamental frequency without noise are calculated from the intact, healthy and damage states of the structure, and 50 samples of state characteristics *D*^*r*^_*i*,*i*+1_, *D*^*u*^_*i*,*i*+1_, *D*^*d*^_*i*,*i*+1_ are obtained from Eqs. (6) and (7). The PDFs of *C*^*u*^_*i*,*i*+1_ and C^*d*^_*i*,*i*+1_ for damage case 1 are calculated from Eqs. (8) and (9), as shown in Fig. 4. The distributions of the two indicators are significantly different for partial structures 1 and 2. This is because spring *k*_2_ within partial structures 1 and 2 is damaged in this scenario.

**Figure 5:**
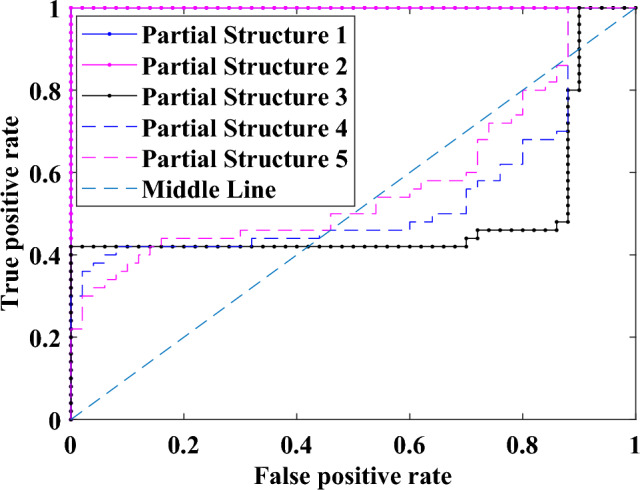
ROC curves of the probability distributions of the damage indicators for damage case 1.

The ROC curve of the PDFs of the damage indicators for damage case 1 is shown in Fig. 5, with the vertical axis representing the true positive rate and the horizontal axis representing the false-positive rate^30,32^. It is noted^33^ that when the ROC curve is close to the upper left corner of the figure, the two PDFs considered are farther apart, indicating a greater likelihood of damage occurrence at the location considered. Conversely, if the ROC curve is close to the diagonal straight line dissecting the figure, the two PDFs are similar, with a small difference in the mean values. This corresponds to a very small likelihood of damage occurring at the corresponding location. The enclosed area in the corresponding AUC can be used to quantitatively measure the likelihood of damage occurrence. If the ROC curve overlaps with the upper left corner, the AUC value equals 1.0. If the ROC curve overlaps with the middle straight line, the AUC value equals 0.5.

**Figure 6:**
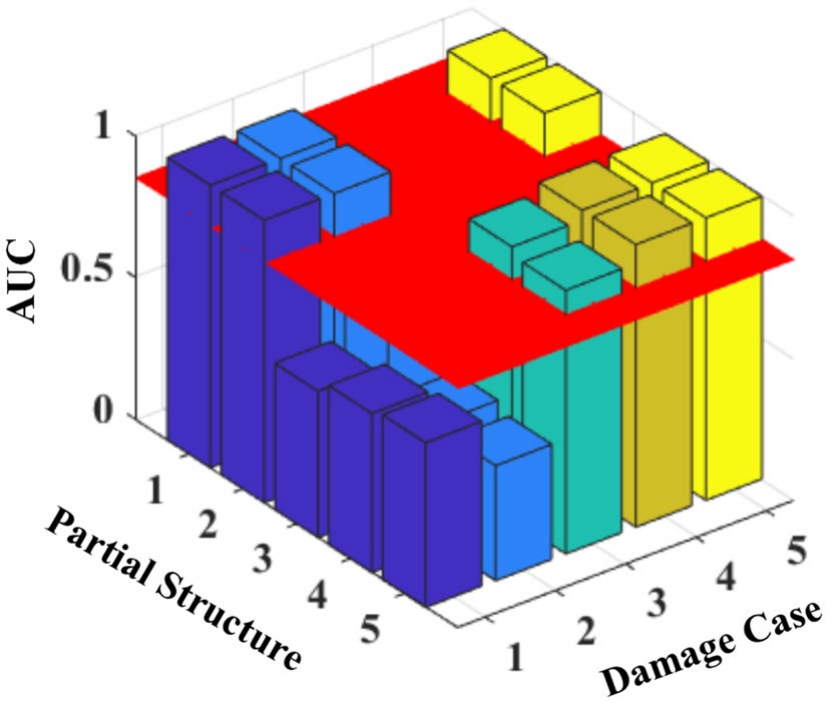
AUC values for different damage cases.

The ROC curves of partial structures 1 and 2 in Fig. 5 almost overlap with the upper left corner, and those for other partial structures vary around the diagonal line. The AUC values from these curves are calculated and shown in Table 1 and Fig. 6. The AUC values of the partial structures, including the damage elements, are much greater than 0.85^32^, indicating the presence of damage in these partial structures. Similar observations are noted in the results of all damage scenarios studied, with AUC values associated with damage approaching unity, whereas other values not related to any damage are approximately 0.5^32^.

**Table 1:** AUC values for different damage cases.

Partial Structure	Elements	AUC values				
		Damage Case 1	Damage Case 2	Damage Case 3	Damage Case 4	Damage Case 5
1	1&2	**0.998**	**0.998**	0.541	0.576	**0.998**
2	2&3	**0.998**	**0.998**	0.541	0.576	**0.998**
3	3&4	0.518	0.739	0.540	0.576	0.823
4	4&5	0.563	0.439	**0.961**	**0.996**	**0.998**
5	5&6	0.583	0.407	**0.931**	**0.998**	**0.998**

Significant values are in bold.

#### Damage detection with noise effects

To consider the influence of measurement noise, white noise *e*_*n*_(*t*) is added to the simulated acceleration responses *ÿ*_*i*_(*t*), and the response signals with noise *ÿ*_*i*_^*n*^(*t*) can be expressed as Eq. (11). The ratio of the noise amplitude to the simulated acceleration (NSA) is set to 0.10, 0.20 and 0.30 for this study^26^.

11 $$ \begin{gathered} \ddot{y}_{i}^{n} \left( t \right) = \ddot{y}_{i} \left( t \right) + e_{n} \left( t \right) \hfill \\ e_{n} \left( t \right) = \delta \times \max \left( {\ddot{y}_{i} \left( t \right)} \right) \times randn \hfill \\ \end{gathered} $$

where δ is the NSA and *max*(•) is the absolute maximum value of the signal. *randn* is a sample from a normal random distribution.

**Figure 7:**
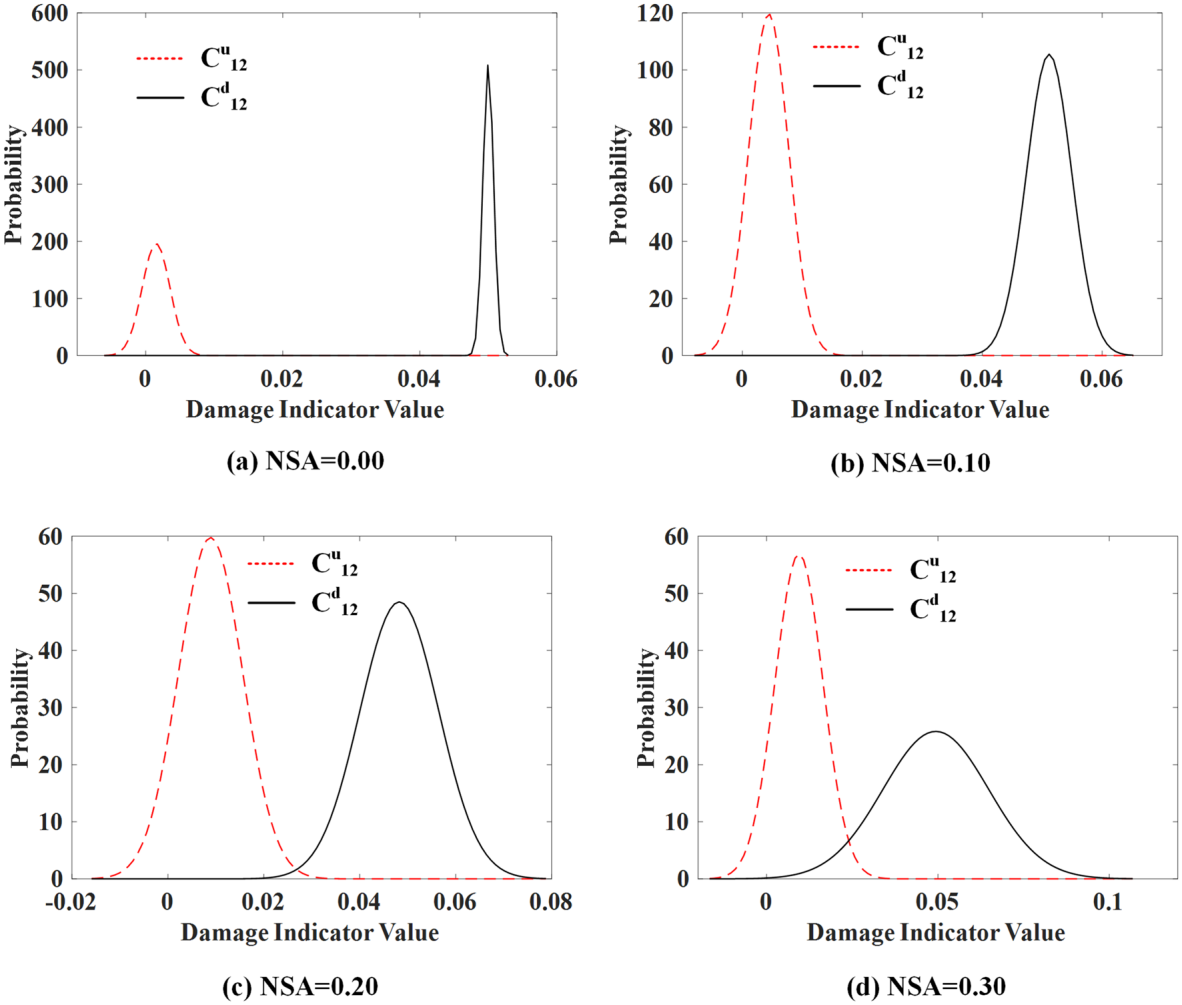
The PDFs of indicators *C*^*u*^_1,2_ and *C*^*d*^_1,2_ for damage scenario 1.

The proposed method is conducted with noise-polluted responses, and the PDFs of damage indicators *C*^*u*^_1,2_ and *C*^*d*^_1,2_ for damage scenario 1 are shown in Fig. 7 for illustration.

**Figure 8:**
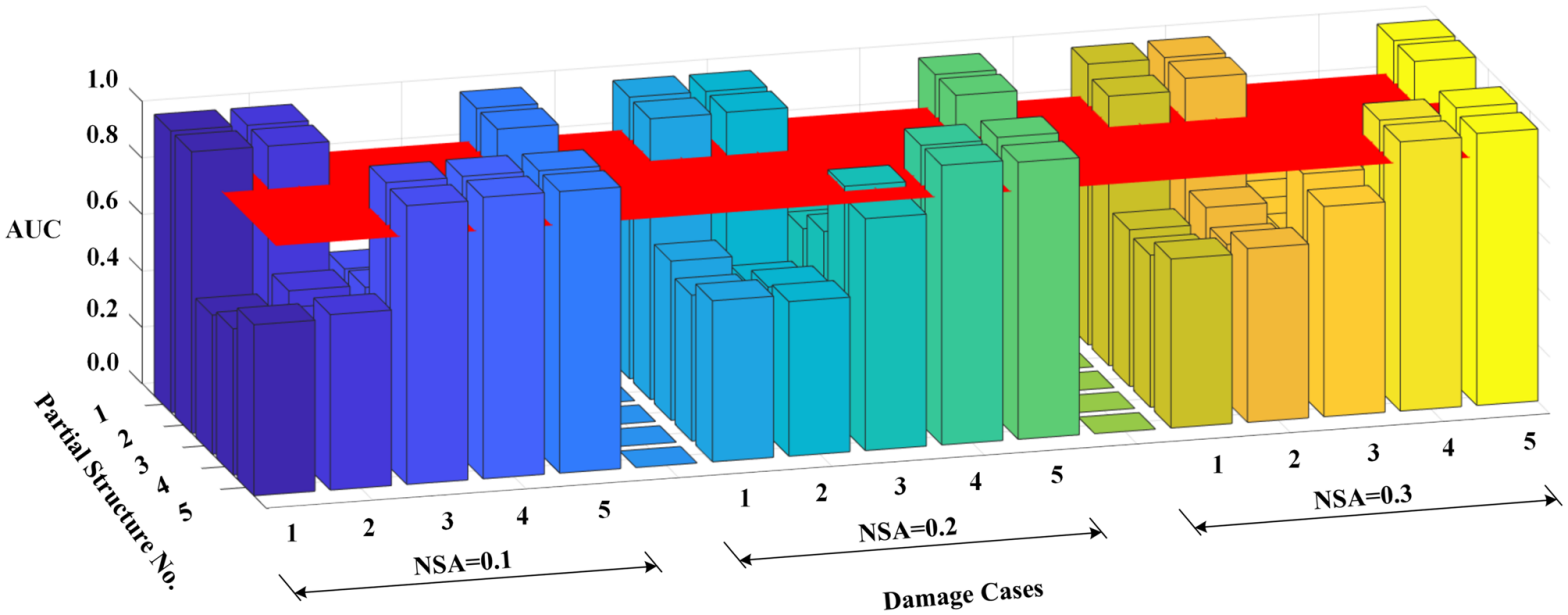
AUC values for different damage scenarios with noise.

The AUC values for different damage scenarios are shown in Fig. 8. The red horizontal plane corresponds to the threshold value of 0.85 for damage occurrence. The values for the partial structures that include the damage elements are much larger than those without any damage, and the AUC values of the damaged partial structures decrease with increasing noise level. For example, the stiffness *k*_5_ is damaged in scenario 3, which affects partial structures 4 and 5. When the noise level increases from 0.1 to 0.3, the AUC values of partial structure 4 decrease from 0.996 to 0.787. The AUC value is above the threshold value of 0.85 when NSA < 0.2, indicating the robustness of the proposed method to measurement noise interference.

### An electricity transmission tower

#### The structure and damage scenarios studied

**Figure 9:**
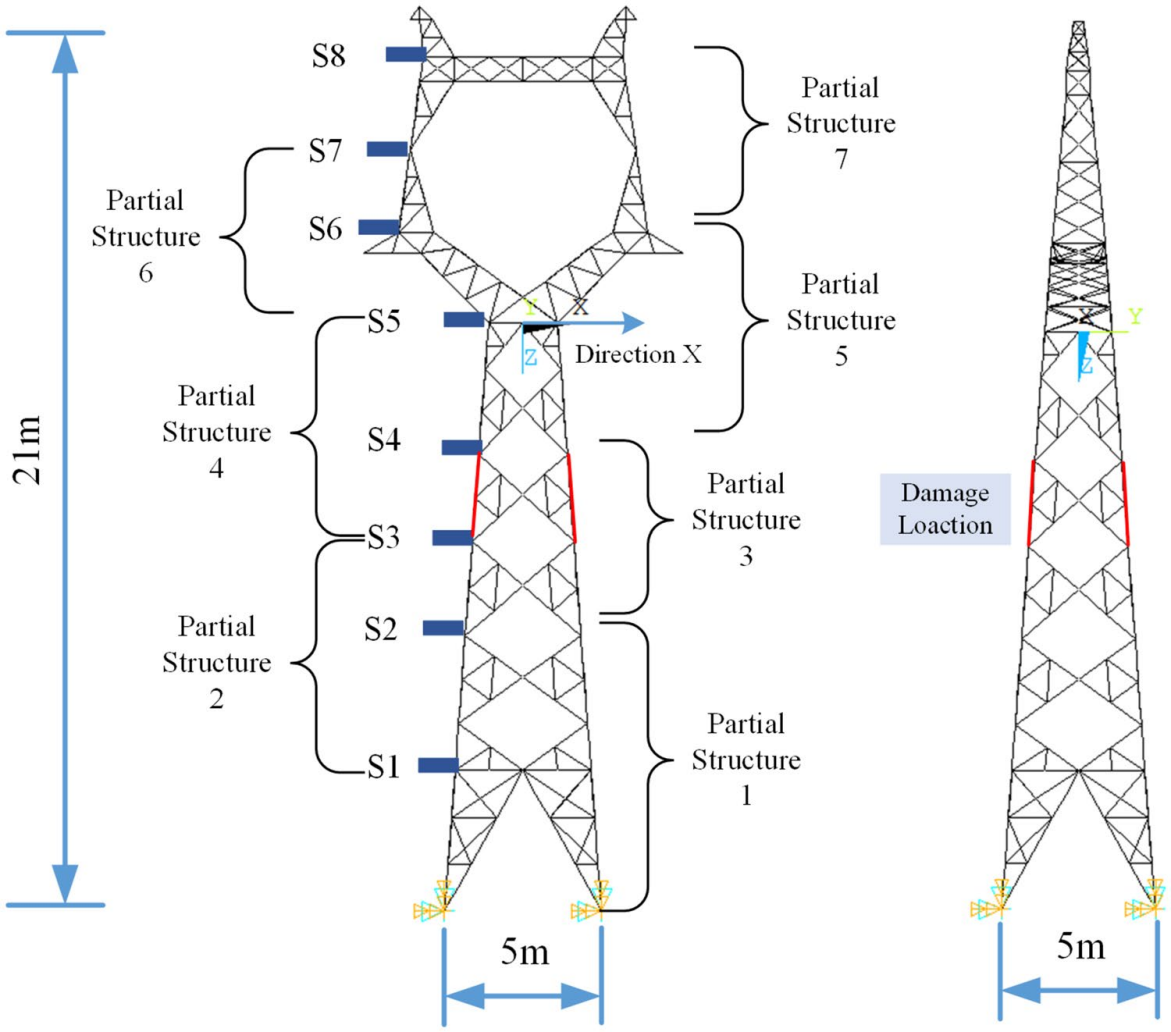
Type 2B3-ZM2 transmission tower and damage scenarios.

A 2B3-ZM2 electricity transmission tower, as shown in Fig. 9, is taken as a typical large-scale structure to verify the effectiveness of the proposed method. The structure is 5 × 5 m in length at the bottom, with a total height of 21 m. It is a lattice structure consisting of many vertical, horizontal and inclined members. Sensors are placed at 8 levels horizontally to collect the dynamic responses under horizontal loading. The structure is divided into 7 partial structures, as shown. Notably, in this example, the proposed method is not applicable for detecting local damage above sensor S8. Local damage to the structure is simulated by reducing the elastic modulus of certain vertical members of the structure, as shown below. The elastic modulus of the materials of four similar vertical members at the same level within partial structure 3 are reduced by 10% (case 1) and 20% (case 2) to simulate two levels of damage. The three-dimensional structure is simplified as a planar structure for this study.

#### Nonstationary excitation and dynamic responses of the tower

To obtain the responses of the tower under nonstationary excitation, a nonstationary fluctuating wind load is assumed, which is obtained via the Kaimal wind spectrum and a normal turbulent wind model based on the IEC61400-1 (Edition 3) code^34^. The turbulence characteristics are set as Class A to simulate a larger fluctuation. The assumed wind load is applied along the x-direction to the two top joints of the tower at the level of sensor S8. The acceleration responses from all 8 sensors along the x-direction are obtained from a finite element model of the structure. All the structural members between the ground level and sensor 2 are included in partial structure 1. For the *i-th* partial structure, all the horizontal and inclined members between sensors *i*-1 and *i* + 1 are included.

**Figure 10:**
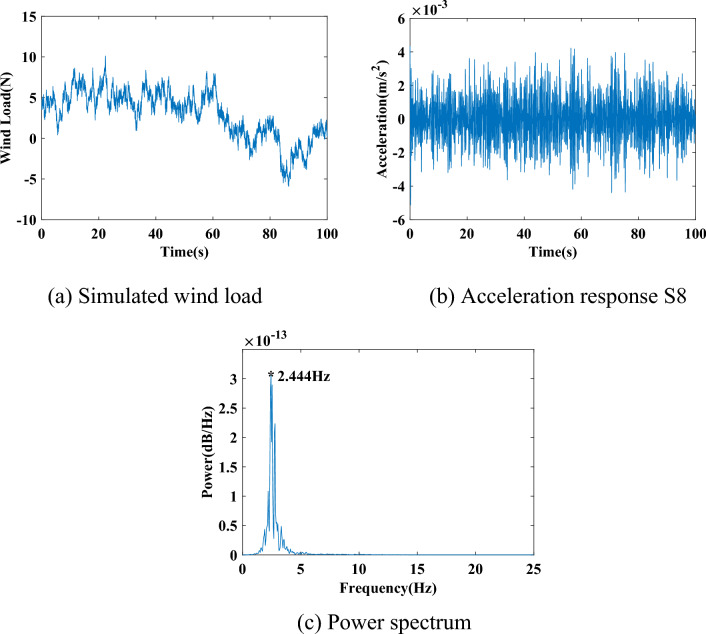
One of the simulated wind loads and acceleration responses S8 under the healthy state.

One hundred sets of data from the healthy state and 50 sets from each damage state are studied. These sets of responses are used to extract the purified GSMTFs and index *D*_*i*,*i*+1_ under different states. Furthermore, to consider the influence of measurement noise, three cases with polluted measurements from different noise levels of 0.10, 0.20 and 0.30 are also studied. The sampling frequency is 50 Hz, and the sampling duration is 100 s. The assumed wind load acting at the level of sensor S8 is shown in Fig. 10(a), and the acceleration response at S8 in the healthy state is shown in Fig. 10(b). The response is noted to exhibit amplitude nonstationarity, with notably larger amplitudes in the middle of the time period.

The power spectral diagram of the acceleration response is shown in Fig. 10(c), which shows the fundamental frequency of the system at 2.444 Hz.

#### Damage detection results

The Gabor transform on the acceleration responses is conducted similar to those in previous studies. Fifty samples of structural responses without noise are simulated for the initial, healthy or damage states of the structure.

With the proposed method, the purified GSMTFs at the fundamental frequency of the structure under initial, healthy and damage states can be determined, and 50 sets of state characteristics *D*^*r*^_*i*,*i*+1_, *D*^*u*^_*i*,*i*+1_, and *D*^*d*^_*i*,*i*+1_ can be calculated. The ROC curve and the AUC values are calculated similarly to those shown in the last example.

**Figure 11:**
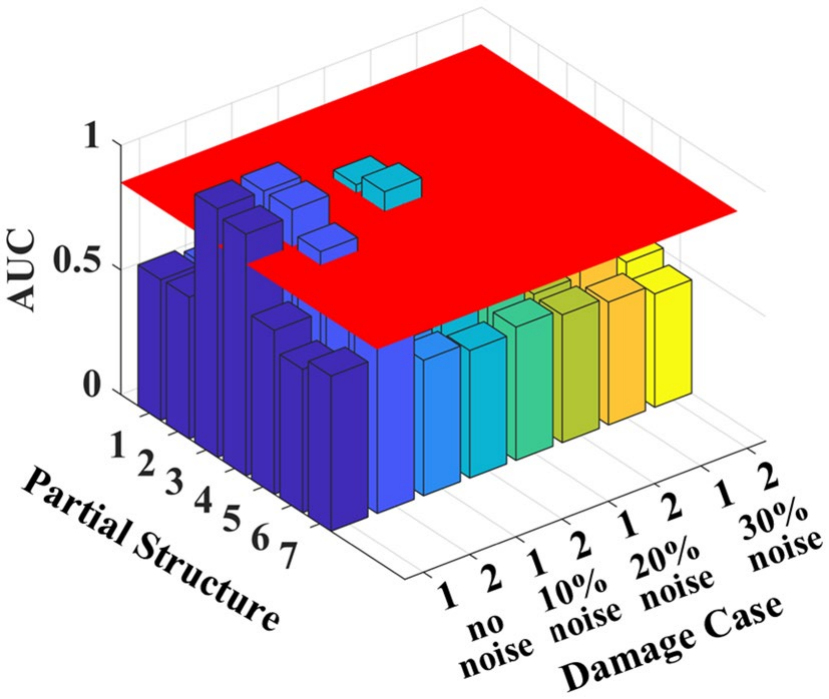
AUC values for different damage cases with different noise levels.

The calculated AUC values are shown in Table 2 and Fig. 11. The partial structure containing the damage elements has much greater AUC values than those without the damage elements. Especially for cases without noise, the AUC values of partial structures 3 and 4 are very close to the maximum value of unity for all damage cases, indicating that the elements in these partial structures are damaged. The identified results are consistent with the simulated damage location. With a 10% noise level, only the damaged partial structures under damage case 2 can be identified, and the lower degree damage under damage case 1 cannot be identified. However, with 10% noise and 20% noise, the AUC values of all the partial structures are less than 0.85, indicating that all the elements are undamaged.

**Table 2:** AUC values for different damage cases with different noise levels.

Partial Structure	Elements	No noise		10% noise		20% noise		30% noise	
		Case 1	Case 2	Case 1	Case 2	Case 1	Case 2	Case 1	Case 2
1	1&2	0.565	0.547	0.546	0.554	0.553	0.517	0.543	0.495
2	2&3	0.569	0.546	0.434	0.496	0.526	0.556	0.562	0.667
3	3&4	**0.998**	**0.998**	0.596	**0.881**	0.448	0.723	0.584	0.527
4	4&5	**0.970**	**0.998**	0.652	**0.927**	0.597	0.692	0.464	0.676
5	5&6	0.658	0.903	0.575	0.618	0.637	0.549	0.518	0.508
6	6&7	0.576	0.699	0.540	0.583	0.627	0.522	0.583	0.508
7	7&8	0.623	0.751	0.544	0.512	0.535	0.513	0.493	0.455

Significant values are in bold.

The results show that noise has an impact on the damage diagnosis of large actual structures. The reason is that the real transmission tower is a lattice structure, and the proposed method in this paper assumes that the cantilevered structure consists of 8 partial structures. For each partial structure, all the vertical, horizontal and inclined members between two adjacent sensors are included, and only the damage to the main chords of the transmission tower is simulated. Therefore, the degree of decrease in the actual lateral stiffness is less than the preset damage degree, and the identification results are more susceptible to noise influence than the 6-DoF spring‒mass system is.

## Laboratory verification

### Steel frame testbed and damage cases

**Figure 12:**
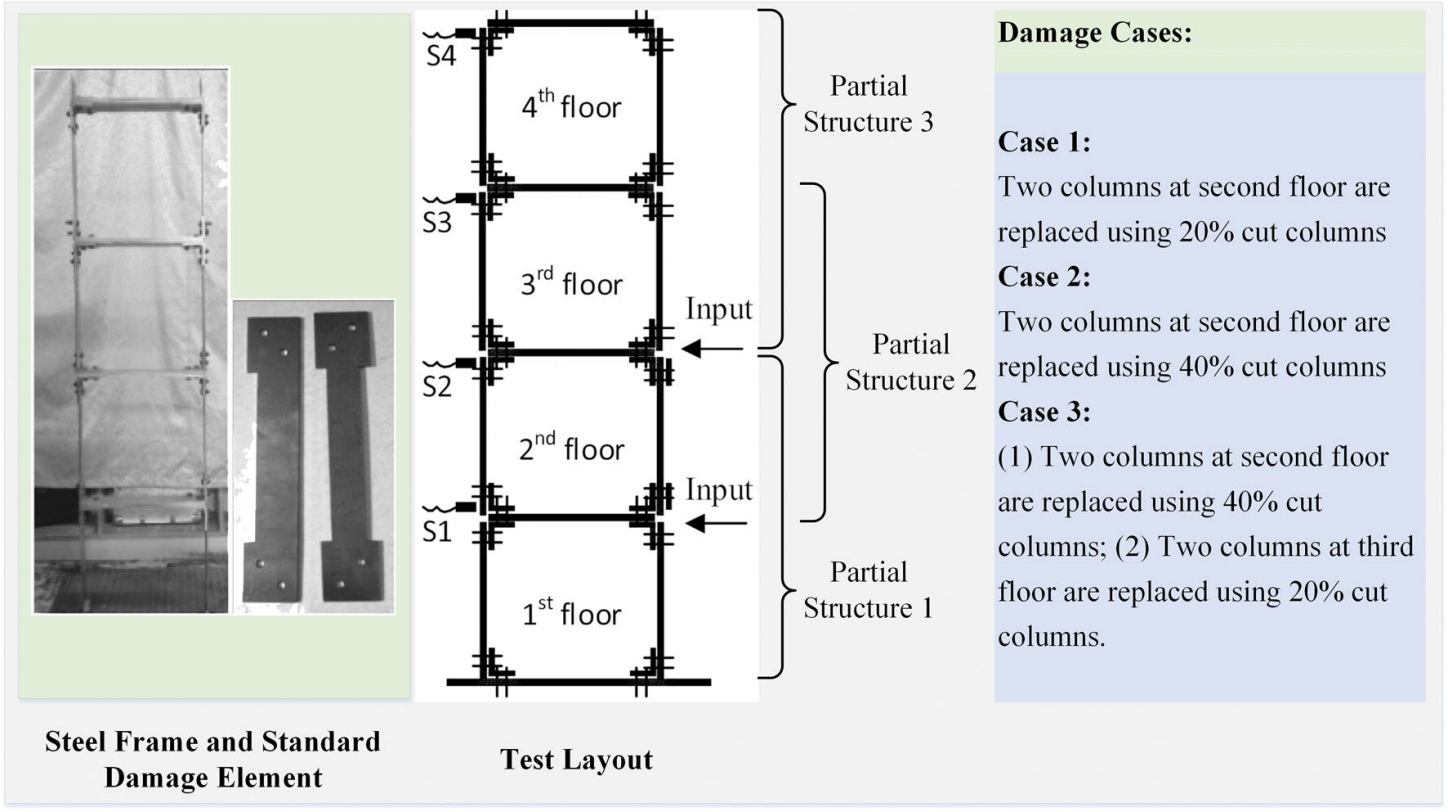
The laboratory steel frame and damage cases.

A planar laboratory steel frame, as shown in Fig. 12, is studied to verify the proposed method. The frame is fabricated from steel plates, gusset plates and bolts. The dimensions of all the vertical steel members are 350 × 65 × 4 mm.

Three damage cases are simulated in the test by replacing the intact vertical member with one whose width is reduced along its full length. Damage member 1 has the width of the member reduced by 20% on one edge, whereas damage member 2 has the width reduced by 20% on both edges. The structure is divided into three partial structures, as shown for damage identification.

### Excitation and dynamic responses

Random taps by two hammers at the first and second floor levels of the frame are used to excite the structure at approximately 0.5 s intervals. The material at the hammer head is soft rubber. The acceleration responses of each floor were measured by YD81D-V accelerometers installed at the joints and collected by a dynamic data acquisition system. The sampling frequency is 150 Hz, and the total sampling duration is 3200 s for measurement in the healthy state. These responses are divided into 80 segments of 40 s each for the estimation of the PDFs of the damage indicators.

**Figure 13:**
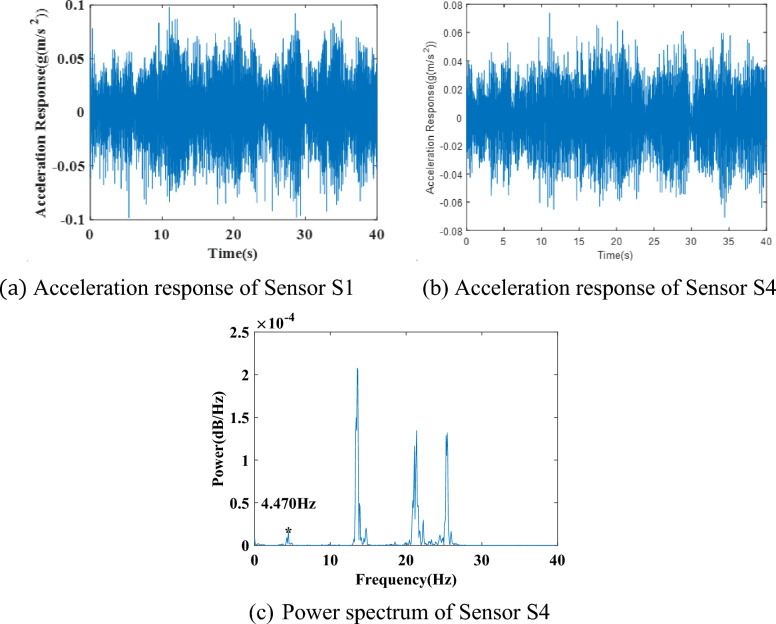
Acceleration responses and their power spectra in the healthy state.

The acceleration responses of sensors S1 and S4 are shown in Fig. 13(a),(b). The power spectrum of the acceleration response at sensor S4 is shown in Fig. 13(c), which shows the fundamental frequency of the system at 4.470 Hz. It is similar to those in the previous study but has 2048 discrete points in the spectrum from the 6000 data points in the data segment of the signal.

### Damage detection via the proposed method

The first 40 segments are used to calculate the purified GSMTFs at the fundamental frequency of the structure and the state characteristics *D*^*r*^_*i*,*i*+1_ in the initial state. The remaining 40 segments are used to calculate the purified GSMTFs at the fundamental frequency and state characteristics *D*^*u*^_*i*,*i*+1_ in the healthy state. Then, the PDF of damage indicators *C*^*u*^_*i*,*i*+1_ in the healthy state can be identified from Eqs. (8) and (9). There are 2048 discrete points in the Gabor transform, with the shape parameter of the Gabor transform equal to 1.537.

For each damage scenario studied, the total sampling time is 1520 s, and 38 data segments are obtained with 40 s each. All 38 segments are used to calculate the purified GSMTFs at the fundamental frequency and state characteristics *D*^*d*^_*i*,*i*+1_ for each damage scenario. Then, the PDF of damage indicators *C*^*d*^_*i*,*i*+1_ for each damage case can be estimated on the basis of the state characteristics *D*^*r*^_*i*,*i*+1_ and *D*^*d*^_*i*,*i*+1_.

**Figure 14:**
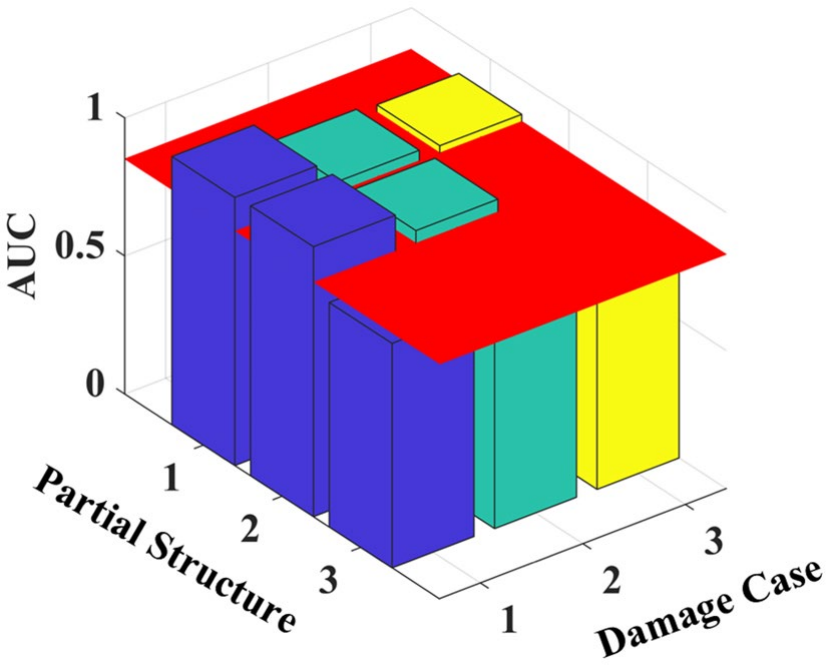
AUC values for different damage cases.

The ROC curves are then plotted from the PDFs of damage indicators *C*^*u*^_*i*,*i*+1_ and *C*^*d*^_*i*,*i*+1_, and the AUC values calculated for each damage scenario are shown in Table 3 and Fig. 14. The results show that the AUC values of the partial structure containing the damage elements are larger than those without the damage elements. In damage cases 1 and 2, only the AUC values of the partial structures containing the damage elements are greater than 0.85, which is consistent with the true location of the damage. For damage case 3, only the AUC values of the partial structure containing the damage element with 40% width reduction are greater than 0.85, whereas those with 20% width reduction at the third floor cannot be identified.

**Table 3:** AUC values for different damage cases.

Partial Structure	Floor	AUC values		
		Damage Case 1	Damage Case 2	Damage Case 3
1	1&2	**0.971**	**0.886**	**0.874**
2	2&3	**0.978**	**0.894**	0.816
3	3&4	0.813	0.713	0.828

Significant values are in bold.

### Comparison with existing dot product difference indices

The dot product difference (DPD) index has also been proposed to identify structural damage on the basis of GSMTFs or TFs at the fundamental frequency^23^. The index has the advantage of scaling between 0 and 1. The DPD index for damage identification can be expressed as Eq. (12) when GSMTFs at the fundamental frequency are used.

12 $$ DPD_{i,j}^{d} = 1 - \left| {\frac{{T_{i,j}^{r} - T_{i,j}^{d} }}{{\left| {T_{i,j}^{r} } \right|\left| {T_{i,j}^{d} } \right|}}} \right|, $$

where *T*_*i*,*j*_ is the GSMTF at the fundamental frequency between the *i-th* and *j-th* DoFs, which is equal to the mean value of GSMTF^*k*^_*i*,*j*_(*a*_1_) (*k* = 1,2,3,4). Superscripts *r* and *d* denote the initial state and damage state, respectively. Similarly, the GSMTFs at the fundamental frequency under initial and healthy states can be used to reduce the influence of noise. The DPD index under the healthy state can be expressed as Eq. (13).

13 $$ DPD_{i,j}^{u} = 1 - \left| {\frac{{T_{i,j}^{r} - T_{i,j}^{u} }}{{\left| {T_{i,j}^{r} } \right|\left| {T_{i,j}^{u} } \right|}}} \right|, $$

where the *s*uperscript *u* denotes the healthy state. Similarly, if *Q* samples of responses of the structure under initial, healthy and damaged states are collected, *Q* samples *T *^*r*^_*i*,*j*_, *T *^*u*^_*i*,*j*_, and *T *^*d*^_*i*,*j*_ can be calculated. Then, *Q* samples of *DPD*^*u*^_*i*,*j*_ and *DPD*^*d*^_*i*,*j*_ can be obtained. Finally, the ROC curve and AUC values can be calculated.

**Figure 15:**
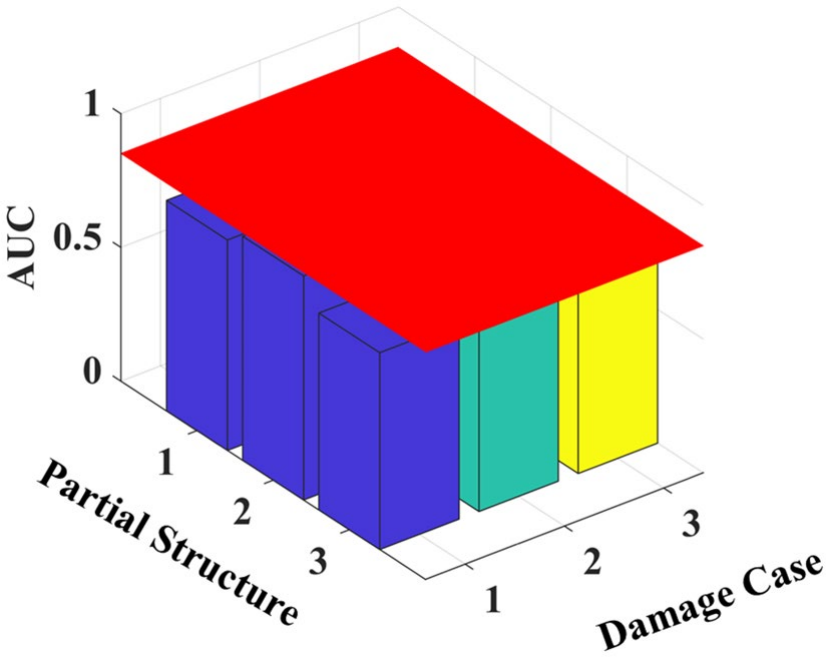
AUC values for different damage cases according to the DPD index.

The mean values of GSMTF^*k*^_2,1_, GSMTF^*k*^_3,1_, and GSMTF^*k*^_4,2_ (*k* = 1,2,3,4) under different structural states are used to calculate the DPD index and AUC values, as shown in Table 4 and Fig. 15. The results show that the AUC values for all partial structures are less than 0.85, making the identification of damage locations difficult. Therefore, the proposed method can better identify damage.

**Table 4:** AUC values for different damage cases according to the DPD index^23^.

Partial Structure	Floor	AUC values		
		Damage Case 1	Damage Case 2	Damage Case 3
1	1&2	0.787	0.787	0.772
2	2&3	0.838	0.733	0.733
3	3&4	0.736	0.698	0.699

## Conclusions

In this paper, a new damage detection method based on the Gabor transform, SVD and ROC curves is developed. A new state characteristic is established on the basis of the purified GSMTFs at the first natural frequency, and a damage indicator is proposed. Furthermore, the PDFs of damage indicators under healthy and damaged states are identified, and the ROC curves and AUC values are used to determine the damage location. Two numerical examples and a plane frame in the laboratory are used to verify the effectiveness of the proposed method.

1. In the six-degree-of-freedom system numerical example, amplitude nonstationary loads are applied. Different damage locations and degrees are considered, and the influence of measurement noise is studied. The results show that the proposed method can identify the damage location and is robust to noise. Even at the 30% noise level, the AUC values of the damaged partial structures are still much greater than those of the other structures.
2. In the transmission tower numerical example, the assumed nonstationary fluctuating wind loads and two damage cases are considered. The results show that the damage locations can be identified successfully. Moreover, because of the simplification of the identification structure, damage identification for minor damage to local components is more susceptible to noise interference, so measurement equipment with an antinoise system should be adopted.
3. In the laboratory test, random percussion excitation is considered, and the damage location and degree are studied. The results show that the AUC values of the partial structure containing the damage elements are much greater than those of the other structures are, and the proposed method can identify the damage location. Moreover, the proposed method is compared with the DPD index, and the results show that the AUC values for all partial structures are less than 0.85, making the identification of damage locations difficult. The proposed method can better identify damage.

However, in the proposed method, the structures are simplified as cantilever structures with several DoFs, and only the damage between three adjacent DoFs can be considered, not for each member. Therefore, the applicability of the proposed method for identifying other types of structures or damage members requires further in-depth research.

## Data Availability

The datasets used and/or analyzed during the current study are available from the corresponding author upon reasonable request.
